# Provision of services in primary care for type 2 diabetes: a qualitative study with patients, GPs, and nurses in the East of England

**DOI:** 10.3399/bjgp20X710945

**Published:** 2020-07-28

**Authors:** Hajira Dambha-Miller, Simon J Griffin, Ann Louise Kinmonth, Jenni Burt

**Affiliations:** Primary Care Unit, Department of Public Health and Primary Care, University of Cambridge, Cambridge; Division of Primary Care and Population Health, University of Southampton, Southampton.; Primary Care Unit, Department of Public Health and Primary Care, University of Cambridge, Cambridge.; Primary Care Unit, Department of Public Health and Primary Care, University of Cambridge, Cambridge.; Healthcare Improvement Studies Institute, University of Cambridge, Cambridge.

**Keywords:** diabetes mellitus, type 2, general practitioners, health services, nurses, primary health care, provision

## Abstract

**Background:**

There is little evidence on the impact of national pressures on primary care provision for type 2 diabetes from the perspectives of patients, their GPs, and nurses.

**Aim:**

To explore experiences of primary care provision for people with type 2 diabetes and their respective GPs and nurses.

**Design and setting:**

A qualitative primary care interview study in the East of England.

**Method:**

Semi-structured interviews were conducted, between August 2017 and August 2018, with people who have type 2 diabetes along with their respective GPs and nurses. Purposive sampling was used to select for heterogeneity in glycaemic control and previous healthcare experiences. Interviews were audio-recorded and analysed thematically. The consolidated criteria for reporting qualitative research were followed.

**Results:**

The authors interviewed 24 patients and 15 GPs and nurses, identifying a changing landscape of diabetes provision owing to burgeoning pressures that were presented repeatedly. Patient responders wanted GP-delivered care with continuity. They saw GPs as experts best placed to support them in managing diabetes, but were increasingly receiving nurse-led care. Nurses reported providing most of the in-person care, while GPs remained accountable but increasingly distanced from face-to-face diabetes care provision. A reluctant acknowledgement surfaced among GPs, nurses, and their patients that only minimum care standards could be maintained, with aspirations for high-quality provision unlikely to be met.

**Conclusion:**

Type 2 diabetes is a tracer condition that reflects many aspects of primary care. Efforts to manage pressures have not been perceived favourably by patients and providers, despite some benefits. Reframing expectations of care, by communicating solutions to both patients and providers so that they are understood, managed, and realistic, may be one way forward.

## INTRODUCTION

There are an estimated 4 million people living with type 2 diabetes in the UK and these numbers are predicted to rise to 4.6 million (9.5% of the population) by 2030.^1^ The rapidly growing prevalence of the disease imposes a large financial burden, with a cost to the NHS of £14 billion a year.^2^ Most diabetes care occurs in general practice, where an average 20 million diabetes contacts occur annually.^3^ This includes, but is not limited to, diabetes annual reviews, personalised care plans, treatment intensification, monitoring for Quality and Outcomes Framework (QOF) indicators, and the day-to-day clinical and social care of diabetes-related complications.^4^^,^^5^ Most people with diabetes will also have multiple morbidities that will require additional primary care services.^6^ National guidelines and QOF indicators require at least annual monitoring and reporting on metabolic markers and both microvascular and macrovascular complications. Universal targets are no longer recommended for achieving tight diabetes control, and there is greater emphasis on more individualised care to avoid overtreatment. However, GP practices were still required to keep a register of people with multimorbidities, where HbA1c levels are ≤59 mmol/mol (adequate control), or if HbA1c levels are ≤64 mmol/mol (poor control), or if HbA1c levels are ≤74 mmol/mol (very poor control) in the preceding 12 months. These specific glycaemic registers will soon be adjusted to reflect frailty levels among people with type 2 diabetes.

Driven by rising workloads and declining resources, the current strain on primary care services has been described extensively.^7^ Labelled a ‘national crisis’, by clinicians and policymakers, there are major concerns for staff wellbeing, recruitment and retention, and the subsequent ability of primary care to provide acceptable quality of care.^3^ The impact of these pressures on care provision for specific conditions, including diabetes, has received less attention. The authors are not aware of any previous studies that have captured patient experiences of primary care diabetes-specific service provision in response to growing pressures.

Patient experiences are important: they provide in-depth and nuanced understanding of current services, as well as being a key component of healthcare quality. Positive patient experiences have been associated with improved safety, clinical effectiveness, and health outcomes.^8^^–^^10^ Patient experience has also been linked to improved doctor satisfaction and wellbeing.^11^ There is a paucity of evidence examining patient experiences alongside those of their respective doctors and nurses. Understanding patient and provider experiences in parallel, within the context of current primary care resource constraints, may help to inform the provision of acceptable and achievable diabetes services.

**Table:** How this fits in

There is limited evidence on the impact of national pressures on primary care provision for type 2 diabetes from the perspectives of patients and their respective GPs and nurses. This interview study revealed increasingly unmet expectations of patient and practitioner aspirations of service delivery. Urgent solutions are needed to reframe expectations, improve staff morale, and encourage more meaningful multidisciplinary task delegation of diabetes care.

This study aimed to explore the perspectives of patients, their GPs, and practice nurses on the current provision of primary care for type 2 diabetes, with a particular focus on how services had changed for them over time.

## METHOD

This was a semi-structured qualitative interview study nested within the 10-year follow-up phase of the Anglo-Danish-Dutch Study of Intensive Treatment in People with Screen Detected Diabetes in Primary Care (ADDITION-Cambridge) trial. ADDITION-Cambridge is a cluster randomised controlled trial of a population-based screening for type 2 diabetes followed by intensive multifactorial treatment compared with routine care.^12^ The trial was conducted in the East of England and recruited 867 people with type 2 diabetes in 49 general practices across urban, suburban, and rural areas. A detailed description of the trial has been reported elsewhere.^13^^,^^14^

### Recruitment and sampling of people with type 2 diabetes

All 867 ADDITION-Cambridge responders within the 10-year follow-up phase of the trial were eligible to participate. These responders were sampled purposively, taking into account reported previous experience of primary care. Experience reports were completed by responders based on the Consultation and Relational Empathy (CARE) measure of holistic and patient-centred care at the 1- and 10-year follow-up phase of the trial.^13^ The CARE measure provides a numerical score of experience (from 10 to 50); equal proportions of responders who reported high (>45), moderate (35–45), and poor (<35) experiences were invited for interview.^15^ Within these groups, responders were also sampled to include a range of glycaemic measures (at the 10-year follow-up phase of the trial), using categories of HbA1c level (≥8.5% [69.4 mmol/mol], 7.5–8.5% [58–69.4 mmol/mol], or ≤7.5% [58 mmol/mol]). Responders were not separated by original trial group arms as the authors have previously reported there were no significant differences in CARE scores between groups, and the trial itself reported no significant reductions in the incidence of cardiovascular events and death.^13^ Invitation letters were sent to all sampled responders, along with a study information leaflet, consent form, and a pre-stamped return envelope. Non-response was followed up with a reminder letter 3 months later. Responders were offered an interview at a mutually convenient location or over the telephone, according to their preferences.

### Recruitment and sampling of GPs and nurses

GPs and nurses were sampled from general practices with patient responders enrolled in the ADDITION-Cambridge trial who had also been recruited to this interview study; the researchers tried to match GP/and or nurses from practices where patient responders had been recruited. Invitation letters were sent to potential responders, with a study information leaflet and consent form. Non-response was followed up by an email reminder. Those who responded were offered an interview at a mutually convenient location, or over the telephone, and were reimbursed for their time.

### Data collection

Interviews were conducted between August 2017 and August 2018. A semi-structured approach was used whereby a topic guide enabled similar areas to be covered across interviews, but interviewers were alert to the need for a flexible approach to ensure that any related subjects of importance could be raised (see Boxes 1 and 2 for topic guide summaries for patients and practitioners). All interviews were audio-recorded (with consent), transcribed verbatim, and subsequently anonymised. A sample of transcripts was checked against the audio-recording to ensure accuracy. Interviews were stopped once consensus had been reached among the study team that there were sufficient quality and depth-of-interview data to inform analysis.^16^ Interviews with people who had diabetes lasted between 45 and 60 minutes while GP and nurse interviews were between 16 and 30 minutes.

**Box 1.** Summary of initial topic guide used to aid questions during interviews with patients

Introductions, explanations, and consent. Experience from diagnosis to present day — chronological sequence in own words. Who looks after diabetes? How is diabetes care set up locally? What has been helpful or unhelpful? What would you like to see in future diabetes care? Long-term experiences. Tell me about your experiences of diabetes care over time? What has changed?

**Box 2.** Summary of initial topic guide used to aid questions during interviews with practitioners

Introductions, explanations, and consent. Describe in your own words how diabetes care is set up locally. Describe your last few diabetes consultations. Barriers and facilitators to care. Could you talk me through some examples of diabetes care that you feel went well? Why did they go well? What could be improved? What could be helpful in caring for diabetes patients in the community? What works well at present?

### Data analysis

The analysis was inductive, in line with qualitative principles, and drew on thematic analysis approaches.^17^^,^^18^ Following the initial three interviews, a first descriptive account of data was generated and discussed within the research team and with responders to enable reflection on the topic guide and sampling strategy before additional data collection. Close reading, re-reading of initial transcripts, and discussion among the team generated a coding framework (refined as data collection progressed) that was used to code the remaining transcripts. Memos were used to help summarise and synthesise codes into themes, which brought related codes together. Particular attention was paid to searching for alternative or outlying perspectives as the interpretation of data progressed. At the end of the interviews, responders were sent a summary of analytical themes with the option to comment or discuss these further.^19^ QSR NVivo software (version 10) was used to code, organise, and manage data. The Consolidated Criteria for Reporting Qualitative Research (COREQ) checklist was used to guide the reporting of findings.

### Data availability

The datasets generated and analysed during the current study are not publicly available. They contain information that could compromise research responder privacy/consent but may be available from the corresponding author on reasonable request.

## RESULTS

The authors interviewed 24 people (11 female; 13 male) with type 2 diabetes (Table 1) all of whom were diagnosed >10 years earlier. Additionally, nine nurses and six GPs from the practices of patient responders were interviewed (Table 2). Of the interviews with patient responders, 14 were carried out in their homes, and 10 by telephone; of the GP and nurse interviews, 10 interviews were carried out at the responders’ workplace, and five by telephone.

**Table 1.** Characteristics of interviewed patients with type 2 diabetes

**Characteristic**		***n* (%)^a^ (*N* = 24)**
**Age, years, mean (SD)**		61 (7)

**Sex**		
	Male	13 (54)
	Female	11 (46)

**Ethnicity**		
	White	19 (79)
	Asian	1 (4)
	Other	4 (16)

**CARE measure**		
	High	12 (50)
	Average	6 (25)
	Poor	6 (25)

**HbA1c group, %**		
	≥8.5	7 (29)
	7.5–8.5	9 (38)
	≤7.5	8 (33)

a Unless specified otherwise. CARE = Consultation and Relational Empathy. SD = standard deviation.

**Table 2.** Characteristics of interviewed GPs and nurses

**Participant characteristic**		***n* (%) (*N* = 15)**
**Sex**		
	Male	5 (33)
	Female	10 (67)

**Ethnicity**		
	White	10 (67)
	Asian	2 (13)
	Other	3 (20)

**Practitioner type**		
	GP	6 (40)
	Nurse	9 (60)

### Summary of findings

The authors identified a changing landscape of diabetes service provision in primary care owing to burgeoning pressures that were presented repeatedly by patients and their respective GPs and nurses. Patient responders wanted GP-delivered care with greater continuity. They saw GPs as experts best placed to support them in managing their condition, but were increasingly receiving nurse-led diabetes care. Nurses reported providing most of the in-person care, while GPs remained accountable, but increasingly distanced from face-to-face provision. A reluctant acknowledgement also surfaced among providers and their patients that only minimum care standards could be maintained, with aspirations for high-quality care provision unlikely to be met. Each of these issues are considered in depth next in this article.

### What patients want: GP-delivered diabetes care

Most patient responders gave broadly positive accounts of diabetes services in primary care. Long-term relationships with GPs and interpersonal care were highly valued:

*‘I like to see the doctor* […] *it’s just that you build up a rapport with the person over the years; you get to know them, you get to know how good they are* […] *that’s all one can ask.’*

(Responder [R1-3], male [M], HbA1c of >8.5%)

Reflecting how patients particularly valued in-person and frequent contact with the GP, they repeatedly expressed the need for more GP consultations for their diabetes care. To them, the GP acted as a physical anchor, providing security and continuity from the time of new diagnosis throughout the long course of their diabetes condition:

‘I wish the doctors could see me more often.’

(R1-13, M, HbA1c of <7.5%)

The need for GP-specific contact was expressed through common descriptions, such as *‘he is all I need’* (R1-4, M, HbA1c of >8.5%) or ‘*I have faith in him’* (R1-9, M, HbA1c <7.5%).

The recently reduced interactions with the GP could lead to feelings of *‘abandonment’* and *‘neglect’* (R1-10, F, HbA1c of <7.5%) for some patients, which became more apparent as the flurry of contact around a new diabetes diagnosis gave way to a more standardised schedule of review appointments:

‘My doctor is my support person. I only need my doctor, nothing else really.’

(R1-17, M, HbA1c of >8.5%)

*‘They see you a lot early you know but now I’m on my own you know, I mean abandoned but I don’t mean like a child, I mean like they don’t want to know me* […] *I would like to see them more.’*

(R1-19, M, HbA1c of 7.5–8.5%)

Responders aged >65 years who had lived with diabetes for many years speculated that recent ‘disappointing’ experiences of care, with a loss of regular contact with their GPs, may be due to their increasing age or length of illness (see Box 3):

‘Once you turn 60 to 65, they want less to do with you, they just tell you over the phone or you ring up.’

(R1-1, M, HbA1c of >8.5%)

‘They don’t care about diabetes once you have had it for a bit. They used to call me more before.’

(R1-6, female [F], HbA1c of 7.5–8.5%)

**Box 3.** Patient aspirations for care

Responder 1-14 (R1-14) is a female aged 73 years with an HbA1c level of <7.5%. The patient had moved GP practices a few times since being diagnosed with type 2 diabetes 13 years ago. This responder described regular telephone and in-person consultations related to diabetes at least every 6 months. This used to include a 30-minute consultation with the nurse for *‘getting ready’* to see the GP including *‘all the foot tests and checks’.* This was followed immediately afterwards with a GP review *‘to make any changes and talk about my diabetes’*. Soon after diagnosis, she attended a *‘diabetes class’* to support her with *‘eating the right things and shopping the right things’*. There were also peer-support meetings that she described as *‘helpful to see what everyone else is doing’* and was given *‘some books with recipes’* that she still uses after more than a decade, though the responder acknowledged that *‘these might be out of date with the new stuff’.* More recently, responder R1-14 described her experiences of diabetes services as *‘hit and miss’*, as she had not seen a GP in 2 years. Most of R1-14’s care is now delivered by nurses but she would prefer to see the GP, though she feels that *‘he isn’t interested in me as I’m too old’.* Responder 1–14 said; *‘I look after my own diabetes’.* The responder explained that after the initial interest early during the disease, *‘they* [GPs] *just decided to leave me to it’.* The responder would like more *‘of the things that we had at the start’* and feels ‘*forgotten because I am getting on in age’*.

### What patients get: nurse-delivered but GP-led diabetes care

Patients, nurses, and GPs all acknowledged that in primary care the nurses have the most face-to-face diabetes patient contact. The nurses described their services as *’limiting the burden on GPs by completing the necessary templates*’ and by ‘*seeing most of the diabetes patients.’* (R2-7, F, nurse)

The GPs did not regard the work undertaken by nurses as replacing that offered by GPs, but suggested that these were ‘supplementary’ or ‘additional’ roles, though the relative contribution and work of GPs and nurses varied between practices:

‘I’m a nurse so most of it is done by us, so they come for their annual review and we adjust the medication up and down according to what the doctor has told us to do, we do the education of diet and exercise, we monitor the blood pressure, cholesterol, make sure they’ve done their urine samples. So, we’ll see them annually unless we feel that things are not under control and then obviously, we make a judgement as to whether we see them 3 months or 6-monthly.’

(R2-15, F, nurse)

‘The nurses do the pre-planning work with blood tests and pre-assessment, and all three of them do diabetes but none of them are specialist nurses in diabetes. I have to do a lot of the main work looking at the medication, looking to see if they need a GP appointment, see if they need a change in medication from that appointment. I do a sort of diabetic virtual clinic, so when we see results coming in that we know the person’s struggling, not getting the medications that we’re using.’

(R2-11, M, GP)

For patients, consultations with nurses were viewed as ‘preparation for seeing the GP’ or, more commonly, as ‘routinised’ or ‘checklist’ consultations following a strict schedule of review appointments, reflected in the passive language patients commonly used when describing such encounters:

*‘Oh* [the nurse] *doesn’t bother talking to me about it and we don’t have a conversation like I do with my doctor. She just follows her script.’*

(R1-19, M, HbA1c of 7.5–8.5%)

*‘I go twice a year and see the diabetic nurse, she only takes the blood, ticks all the boxes that need to have tested and includes the PSA at the same time, so that they get everything checked, take a urine sample and then, as soon as they come back from* [hospital]*, which is usually about 10 days, I’ve already made the appointment to go and see the doctor who can treat me properly and knows my diabetes.’*

(R1-11, M, HbA1 of 7.5–8.5%)

As illustrated above, sharp distinctions were often drawn by patients between the diabetes care provided by GPs and nurses. GPs were portrayed as the ‘experts’, and patients assumed that consulting with them would mean better care. Patients did not express the same level of confidence in nurses and emphasised that ‘ultimate responsibility’ for their diabetes care rested with the GP:

*‘I see the nurse mainly about my diabetes but it’s the doctors who are the professionals. If there are any questions about medicines or things, it’s the doctor really … we need to check with the doctor if she* [nurse] *tries to change things*.*’*

(R1-1, M, HbA1c of >8.5%)

Like their patients, GPs viewed themselves as the experts who held clinical responsibility for decision making on diabetes care. Nurses, too, placed responsibility for diabetes care with the GP, while recognising that nurses predominantly delivered face-to-face care:

*‘Yes, the nurse is doing the checks, you know, the foot check, and having the discussion with the patient in our practice* […] *it would appear to the patient that it is led by the nurse because the patient is having the direct contact with the nurse but it’s the GP that has to look through everything behind the scenes to instruct us.’*

(R2-7, F, nurse)

### How services are adapting: increasing pressures on care

Patients’ narratives frequently recognised demands and growing pressures on services. Their accounts outlined *‘problems with the system’* (R1-19, M, HbA1c of 7.5–8.5%) due to *‘insufficient NHS funding’* (R1-5, M, HbA1c of >8.5%), and suggested their diabetes care was more *‘rushed’* (R1-9, M, HbA1c <7.5%) due to decreased availability and regularity of in-person contact (Box 4):

*‘The doctor was rushing, he just didn’t want to know* […] *but it wasn’t like that before with him. So that was like the start of me losing confidence in the doctors now, you know because they can’t cope with how much he has to do.’*

(R1-10, F, HbA1c of <7.5%)

**Box 4.** Adapting to pressures on services

Responder 2–14 (R2-14) is a male GP. He has cared for patients with type 2 diabetes for 19 years at the same practice, where diabetes services are *‘mainly nurse led’.* Responder 2–14 explained that *‘historically’* they reviewed every patient with diabetes in person, but as the population has grown they have had to *‘let go’* and *‘make way’* for multidisciplinary staff, though continue to have *‘oversight’.* The practice nurses and healthcare assistants review the patients with diabetes and would *‘alert’* R2-14 if there were any *‘complex patient who needs a GP input’.* This will often include discussions about medication changes or further referrals. Responder 2–14 felt that these reviews still *‘take up too much time’* and is considering extra training for the practice nurses to *‘free-up’* *the* GPs’ availability for other tasks. Recently, R2-14 had been trying out *‘virtual clinics’* in which he reviews the records and blood results without the patient present and is able to electronically record a plan for the nurses to relay to the patients when they attend. Responder 2–14 feels that this is probably the *‘most efficient way of keeping an eye on the patients albeit unknown to them’.* The GP also explained that they have had to be flexible with new approaches to care otherwise they would *‘drown in chronic diseases’.* Responder 2–14 further describes the increasing number of patients with type 2 diabetes as *‘overwhelming’* because of the *‘associated never ending administrative and payment tasks’.*

GP and nurse accounts corroborated those of patients, also flagging concerns around time pressures and increasing diabetes workloads. They too expressed concerns about services reaching *‘capacity’* (R2-4, Male, GP). As a result, many had evolved coping strategies to deal with high numbers of patients and limited resources, commonly reducing the time made available to see patients with diabetes in person (Box 4):

*‘Basically, because diabetes is an epidemic* […] *we’ve got to fit lots more patients into less time, because of the ageing population we’ve also got booming other chronic diseases as well, COPD, heart failure, so it’s just trying to manage the workload and patients*’ *expectations and keeping ourselves sane as well. It‘s not really working.’*

(R2-3, F, GP)

‘We don’t have the time to see them all the time so we see them a lot only when they are new.’

(R2-7, F, nurse)

### Impact: adequate but not outstanding care

GPs repeatedly emphasised the *‘impossibility’* (R2-11, M) of delivering optimal diabetes care and suggested that there was a growing acceptance of *‘good rather than excellent.’* (R2-3, F) care They explained that this was because of *‘unmanageable’* or *‘unachievable workloads’* (R2-3, F) and articulated the need for greater funding and workforce support.

Their accounts suggest a demoralised workforce who describe themselves as *‘uninspired’* (R2-11, M) (Box 5):

‘Like all surgeries it’s not always possible to get the best control in our diabetes patients. We’re always rammed for appointments anyway so we can’t even get them back, so we have to just accept a good control rather than excellent control because we can’t actually see them again.’

(R2-3, F, GP)

*‘We just tick the box quick to say that we are monitoring them. I don’t see them as I do a virtual clinic ahead of the nurse appointment. That’s OK, it’s enough but there is no chance to ask them more about what’s going on in* [their] *life.’*

(R2-11, M, GP)

**Box 5.** Adequate rather than outstanding care?

Responder 2–14 (R2-14) is a male GP who has looked after patients with type 2 diabetes for 8 years in two different GP practices. When asked about the local set-up of diabetes care, R2-14 began by explaining that *‘there isn’t enough staff and not enough care’.* Responder 2–14 suggested that his practice has a high proportion of patients with poorly controlled type 2 diabetes, which they found *‘pretty much impossible to sort out’* because of the lack of resources and restrictions on his time. Responder 2–14 explained that they do not have the *‘ability to see everyone in detail’,* describing himself as being ‘*stressed by the work’* and without much *‘higher-level support’.* Responder 2–14 *‘struggles to get all the QOF boxes ticked’* and this has meant reduced funding to their practice, which further restricts resources. Responder 2–14 found the *‘cycle rather exhausting’* and hoped that the *‘government gets a grip’* with extra support to diabetes services.

## DISCUSSION

### Summary

In this study the authors explored experiences of people with type 2 diabetes and their respective primary care professionals, in receiving and providing services in response to current pressures. Responders identified an enduring set of increasingly unmet expectations and wishes from patients with greater nurse-led, protocol-driven care and less GP in-person provision. Accountability for disease management remained with GPs who are increasingly overstretched. Examples of patients feeling abandoned and neglected and doctors accepting lower care standards were particularly worrying, with potential consequences on the risk of diabetes complications and subsequent impact on patient services and costs.

### Strengths and limitations

The inclusion of patients, their respective GPs, and nurse responders is a strength of this study, allowing a complete perspective on the provision of diabetes services. Interviews enabled detailed probing and prompting to elicit views on the topic. Purposive sampling of patients ensured heterogeneity in care experiences and disease severity, while the sample of providers represented different practices, professional backgrounds, and levels of experiences. Most patient responders were of white ethnicity, which reflects the local demographic of the area, but it meant that a detailed account of experiences from minority ethnic groups was not possible. The study was also reliant on patient and healthcare professional responders opting into interviews; this self-selection may have influenced findings as patient responders were likely to reflect more engaged service users, possibly more disgruntled patients, while providers reflected those who were motivated to find time to participate in an interview.

All the included practices were research active and part of the larger ADDITION trial, which may also have influenced patient or practitioner experiences, and subsequent narratives that were provided. It is possible that the present results may not reflect the full scope of diverse views from nurses, GPs, and patients with diabetes, though the researchers did look for alternative perspectives. Responders were interviewed by telephone or in person. The content and quality of data may vary between these different interview modes. The provider interviews were relatively short in comparison with patient interviews. The duration was led by providers, and longer, face-to-face interviews outside of the busy clinical setting may have provided richer data collection.

### Comparison with existing literature

In this study the authors report increasing dissatisfaction arising from unmet patient, GP, and nurse expectations of diabetes services. This is consistent with national trends in primary care, with the latest results from the British Social Attitudes survey reporting that patient satisfaction with general practice services had dropped to 63%, the lowest level since the inception of the national survey in 1983.^20^ This is concerning: health services that do not meet patient expectations result in lower ratings of trust, adherence, and poorer health outcomes, driving increasing burden on services and costs.^8^^,^^21^

From the patient perspective, it appears that some of this dissatisfaction stems from the delegation of GP tasks to multidisciplinary staff. This national strategy is aimed at managing primary care pressures arising, in part, from the growing prevalence of chronic disease in patients and shortfalls in the GP workforce.^22^^,^^23^ The impact of such delegation may be varied; systematic review evidence suggests that consultations with nurses can deliver equivalent health outcomes, and higher patient satisfaction, compared with those with GPs.^24^ However, recent analysis of patient experience data from the national GP Patient Survey shows that patients who wished to see a GP, but instead saw a nurse, had lower levels of confidence and trust in the nurse, and poorer reported communication.^25^ This is reflected in the present findings, where many patients still perceived GPs as the only experts in diabetes care, whom they regretted not being able to see as often as they wished. The rapid pace of change in diabetes service delivery in order to manage demand may not have permitted opportunities to reframe patient expectations and bring them on board with current policy. Resolving this will continue to be problematic as the service moves towards more digital consultations and an expansion in the roles of non-medical practitioners as set out by the UK Government’s long-term plan.^23^ The present findings suggest that, as part of managing pressures on primary care, managing patient expectations and including them in the dialogue on national efforts to tackle primary care pressures is essential.

Co-creating a health service in which patients are involved with current strategies will need to be accompanied by equivalent efforts to bring GPs and nurses on board, despite the challenge of increasing workload pressures.

To date, the UK has the lowest number of doctors and nurses per head of the population: *‘In the UK there is one doctor for every 356 people, compared with one for every 277 people on average across comparable countries.’*^26^ Problems of recruitment and retention are well known.^27^ GPs already have the lowest morale of all doctors, and 93% of 16 000 GPs in one survey reported that current workloads were negatively impacting clinical care.^28^ The authors found poor staff morale and aspirations for only minimum clinical care standards for type 2 diabetes. This needs to be addressed urgently by government and practice policy.

### Implications for practice

Type 2 diabetes is a tracer condition that reflects many aspects of primary care, and the present findings are therefore likely to have wider implications. With rising pressures on service provision, patient, GP, and nurse expectations of care increasingly remain unmet.

Efforts to manage pressures in primary care have not been perceived favourably by patients and providers. One way forward is to reframe expectations of care, by communicating solutions to both patients and providers so that they are understood, managed, and realistic. Meaningful delegation of accountability to multidisciplinary staff, and efforts to boost existing staff morale, also have an important part to play in delivering manageable yet impactful solutions.

## Funding

ADDITION-Cambridge was supported by the Wellcome Trust (grant reference number: G061895), the Medical Research Council (grant reference number: G0001164 and Epidemiology Unit programme: MC_ UU_12015/4), the National Institute for Health Research (NIHR) Health Technology Assessment Programme (grant reference number: 08/116/300), NIHR Programme Grants for Applied Research (RP-PG-0606-1259), National Health Service Research and Development support funding (including the Primary Care Research and Diabetes Research Networks), and the NIHR. Simon J Griffin is an NIHR senior investigator. The University of Cambridge has received salary support in respect of Simon J Griffin from the NHS in the East of England through the Clinical Academic Reserve. The Primary Care Unit is a member of the NIHR School for Primary Care Research and supported by NIHR Research funds. Hajira Dambha-Miller is an NIHR academic clinical lecturer and was an NIHR doctoral research fellow (DRF-2015-08-027) during this research. Jenni Burt is supported by the Health Foundation’s grant to the University of Cambridge for Healthcare Improvement Studies Institute. The views expressed are those of the authors and not necessarily those of the NHS, the NIHR, or the Department of Health and Social Care. The sponsor had no role in study data collection, data analysis, data interpretation, or writing of the findings. The corresponding author had full access to all data in the study and had final responsibility for the decision to submit for publication.
